# A Model for Trans-Kingdom Pathogenicity in *Fonsecaea* Agents of Human Chromoblastomycosis

**DOI:** 10.3389/fmicb.2018.02211

**Published:** 2018-10-09

**Authors:** Gheniffer Fornari, Renata Rodrigues Gomes, Juliana Degenhardt-Goldbach, Suelen Silvana dos Santos, Sandro Rogério de Almeida, Germana Davila dos Santos, Marisol Dominguez Muro, Cleusa Bona, Rosana Herminia Scola, Edvaldo S. Trindade, Israel Henrique Bini, Lisandra Santos Ferreira-Maba, Daiane Rigoni Kestring, Mariana Machado Fidelis do Nascimento, Bruna Jacomel Favoreto de Souza Lima, Morgana F. Voidaleski, Douglas André Steinmacher, Bruna da Silva Soley, Shuwen Deng, Anamelia Lorenzetti Bocca, Moises B. da Silva, Claudio G. Salgado, Conceição Maria Pedroso e Silva de Azevedo, Vania Aparecida Vicente, Sybren de Hoog

**Affiliations:** ^1^Microbiology, Parasitology and Pathology Post-graduation Program, Department of Basic Pathology, Federal University of Paraná, Curitiba, Brazil; ^2^Embrapa Forestry, Brazilian Agricultural Research Corporation (EMBRAPA), Colombo, Brazil; ^3^Department of Clinical and Pharmacological Analysis, College of Pharmaceutical Sciences, University of São Paulo, São Paulo, Brazil; ^4^Support and Diagnosis Unit, Mycology Laboratory, Hospital of Clinics, Federal University of Paraná, Curitiba, Brazil; ^5^Department of Botany, Federal University of Paraná, Curitiba, Brazil; ^6^Hospital of Clinics, Federal University of Paraná, Curitiba, Brazil; ^7^Department of Cell Biology, Federal University of Paraná, Curitiba, Brazil; ^8^Vivetech Agrociências, Paraná, Brazil; ^9^Department of Pharmacology, Federal University of Paraná, Curitiba, Brazil; ^10^Department of Medical Microbiology, People’s Hospital of Suzhou National New & Hi-Tech Industrial Development Zone, Jiangsu, China; ^11^Dermato-Immunology Laboratory, Institute of Biological Sciences, Federal University of Para, Marituba, Brazil; ^12^Department of Cell Biology, University of Brasília (UnB), Brasília, Brazil, 13 Department of Medicine, Federal University of Maranhão, São Luís, Brazil; ^13^Department of Medicine, Federal University of Maranho, São Lus, Brazil; ^14^Westerdijk Fungal Biodiversity Institute, Utrecht, Netherlands; ^15^Center of Expertise in Mycology Radboudumc/CWZ, Nijmegen, Netherlands

**Keywords:** *Fonsecaea*, chromoblastomycosis, virulence, *Mimosa pudica*, *Bactris gasipaes*, *Tenebrio molitor*, animal model, plant model

## Abstract

The fungal genus *Fonsecaea* comprises etiological agents of human chromoblastomycosis, a chronic implantation skin disease. The current hypothesis is that patients acquire the infection through an injury from plant material. The present study aimed to evaluate a model of infection in plant and animal hosts to understand the parameters of trans-kingdom pathogenicity. Clinical strains of causative agents of chromoblastomycosis (*Fonsecaea pedrosoi* and *Fonsecaea monophora*) were compared with a strain of *Fonsecaea erecta* isolated from a living plant. The clinical strains of *F. monophora* and *F. pedrosoi* remained concentrated near the epidermis, whereas *F. erecta* colonized deeper plant tissues, resembling an endophytic behavior. In an invertebrate infection model with larvae of a beetle, *Tenebrio molitor, F. erecta* exhibited the lowest survival rates. However, *F. pedrosoi* produced dark, spherical to ovoidal cells that resembled muriform cells, the invasive form of human chromoblastomycosis confirming the role of muriform cells as a pathogenic adaptation in animal tissues. An immunologic assay in BALB/c mice demonstrated the high virulence of saprobic species in animal models was subsequently controlled via host higher immune response.

## Introduction

The fungal genus *Fonsecaea* comprises several etiologic agents of human chromoblastomycosis, a severely mutilating skin disease. The genus belongs to the family Herpotrichiellaceae, which consists of numerous species potentially causing a wide range of recalcitrant infections. Among these are cerebral and disseminated diseases which if untreated mostly lead to the death of the patient (Najafzadeh et al., 2011; Doymaz et al., 2015; Gomes et al., 2016). In general, immunocompromised individuals are more susceptible to fungal infections; however, in black fungi infection is also observed in apparently healthy individuals. Vertebrate hosts other than humans include fish and amphibians, other host animals being very rare (de Hoog et al., 2011).

Chromoblastomycosis is characterized by a chronic involvement of cutaneous and subcutaneous tissues containing the fungal invasive form, the muriform cell embedded in microabscesses and fibrosis. The infection often shows skin tissue proliferation, leading to clinically recognizable nodular, tumoral (cauliflower-like), verrucous, scarring, or plaque-like lesions (Queiroz-Telles et al., 2017). The prevalent agents of the disease include *Fonsecaea pedrosoi, Fonsecaea monophora, Cladophialophora carrionii* (Badali et al., 2008; Doymaz et al., 2015), and *Rhinocladiella aquaspersa* (Badali et al., 2010; González et al., 2013). *Cladophialophora carrionii* is found in arid and semi-arid climates (Lavelle, 1980), while *Fonsecaea* species are endemic to the areas with a warm and humid climate (Najafzadeh et al., 2011).

The causative fungal species seem to be implanted into the host skin through sharp specimens of plant debris, such as thorns, carrying the respective opportunistic agent. In this regard, a report indicated their isolation from plant debris, while non-pathogenic relatives were occasionally derived from living plants (Vicente et al., 2008). Epidemiological data provided evidence of traumatic infection by puncture of plant material (Rubin et al., 1991; Fernández-Zeppenfeldt et al., 1994; Queiroz-Telles et al., 2017). Marques et al. (2006) isolated *Fonsecaea*-like fungi from the shells of babassu coconuts of the palm tree, *Orbignya phalerata* and Salgado et al. (2004) found a species morphologically resembling *Fonsecaea* on the thorns of a *Mimosa pudica* plant, which the patient identified as the possible source of his disease.

However, molecular studies have demonstrated that the major part of the environmental strains morphologically that are similar to clinical strains do not necessarily belong to the same species (Crous et al., 2006; Mostert et al., 2006; Vicente et al., 2013). Sequence data of the clinical strains of *C. carrionii* and of a *Cladophialophora* species isolated from cactus thorns surrounding the cottage of a patient demonstrated them to belong to a different species, *C. yegresii*, which had never been observed to infect a human host (de Hoog et al., 2007). Upon environmental sampling to recover the clinical species (Marques et al., 2006; Vicente et al., 2008, 2013), *F. pedrosoi* and *F. monophora* were only rarely encountered, while the majority of environmental species were not known from human infections. Vicente et al. (2013) described the environmental *Fonsecaea* sibling species as novel taxa, named *F. minima* and *F. erecta*.

The apparent selection by human tissue of *F. monophora* and *F. pedrosoi* remains unexplained. The disease is characterized by the presence of muriform cells inside host tissues, which can also be reproduced *in vitro* in non-pathogenic species (Badali et al., 2008). de Hoog et al. (2007) demonstrated that both the clinical species, *C. carrionii* and the environmental, *C. yegresii* could produce muriform cells upon their artificial inoculation into cactus plants, which seemed to contradict the observed predilections for animal hosts (Vicente et al., 2013). In addition, more detailed understanding of the natural ecology and environmental niches of chromoblastomycosis agents is required to monitor the possible routes of infection (Gümral et al., 2014). Adequate infection models in animal and plant hosts are required to build up plausible evolutionary hypotheses.

Therefore, the present study compared plant and animal models to evaluate the associations of hosts with clinical and environmental *Fonsecaea* strains to understand the mechanisms involved in the adaptation of fungal animal and plant disease to elucidate routes of infection of this implantation disease.

## Materials and Methods

### Strains

The fungal strains were acquired from the reference collection of Centraalbureau voor Schimmelcultures (CBS; housed at the Westerdijk Fungal Biodiversity Institute, Utrecht, Netherlands) and Microbiological Collections of Paraná Network (CMRP, Curitiba, Brazil), i.e., *F. pedrosoi* (CBS 271.37) and *F. monophora* (CBS 102248), both isolated from human chromoblastomycosis, and the environmental species, *F. erecta* (CBS 125763) isolated from a living plant (Vicente et al., 2013). In addition, an endophyte, *Colletogloeopsis dimorpha* (CMRP 1417) isolated from *M. pudica* and a strain of *Cladosporium tenuissimum* (CMRP 1441) isolated as endophyte from *Bactris gasipaes* were utilized as controls. The cultures were maintained on 2% malt extract agar (MEA) and Sabouraud’s glucose agar (SGA) at 28°C.

### Plant Inoculum Preparation

The fungal strains were grown on potato dextrose agar (PDA) at 28°C for 7 days. Conidial production was enhanced by passing the cells through a liquid PDA medium in a shaker at 200 rpm at 30°C. After 5 days, the cells were allowed to settle; hyphae and conidia were decanted, and the supernatant was filtered through a cell strainer membrane with 40 μm pore size. After repeated washings with phosphate buffered saline (PBS), the inocula were adjusted to the concentration tested and the cell viability was determined by plating of the suspensions on Mycosel agar followed by incubation for 7 days at 28 and 37°C for *Fonsecaea* species, while the control strains were incubated on PDA at 28°C for 7 days.

### *In vitro* and in Vessel Assays using *M. pudica* and *B. gasipaes*

Seeds of *M. pudica* were provided by the Brazilian Agricultural Research Corporation-EMBRAPA (Manaus, Brazil). Seeds were washed by keeping them for 30 min in flasks under running tap water, then surface sterilized inside a laminar flow chamber with 70% ethanol for 1 min and 5% sodium hypochlorite (NaOCl) for 10 min followed by rinsing thrice with autoclaved distilled water. The seeds were incubated in concentrated sulfuric acid for 15–20 min followed by rinsing thrice in autoclaved distilled water (Souza Filho et al., 2001; Paiva and Aloufa, 2009). Subsequently, the seeds were cultured in agar-solidified Murashige and Skoog (MS) medium (Murashige and Skoog, 1962) containing 3% sucrose for germination. The seeds began to germinate after 48 h under aseptic conditions. An *in vitro* cultured palm plant (*B. gasipaes*) was provided by Vivetech Agrociências using the protocols based on Steinmacher et al. (2011).

In the *in vitro* and in vessel assays, both plants tested were inoculated with three *Fonsecaea* species (*F. pedrosoi* “Fp”, *F. monophora* “Fm”, and *F. erecta* “Fe”), The endophyte, *C. dimorpha* (CMRP 1417) was utilized as the positive control and sterile saline (NaCl 0.85%) was utilized as negative control. The experiments were performed in duplicate with groups of four plants per evaluated strain.

### *In vitro* Plant Inoculation

**FIGURE 1:**
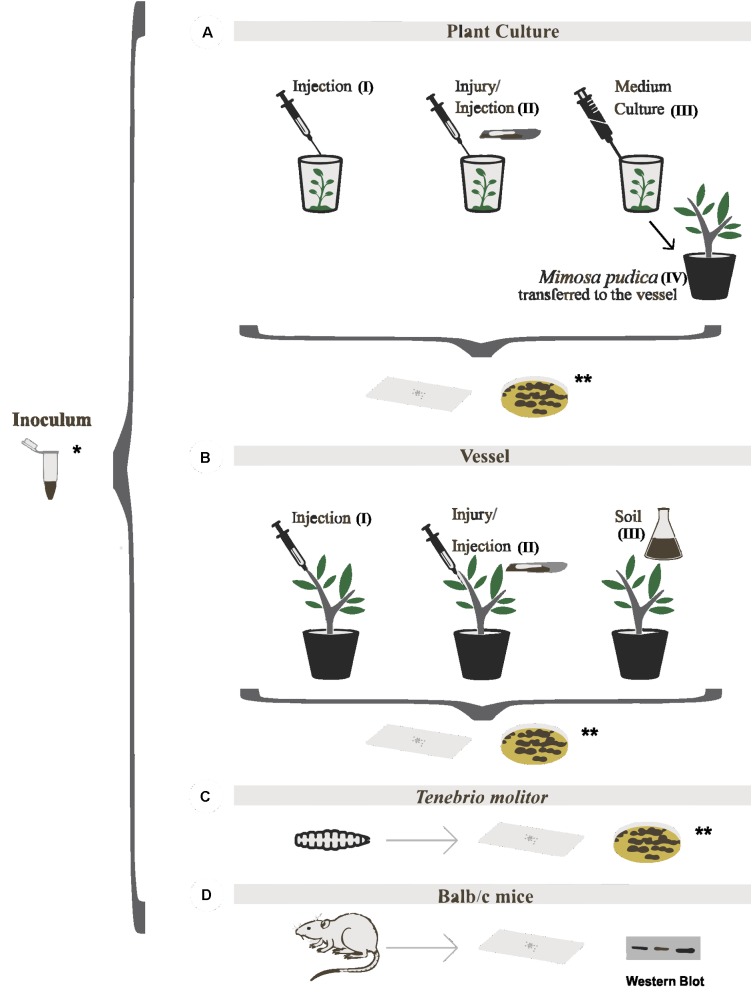
Virulence models tested in *Fonsecaea* sibling species. **(A)** *In vitro* inoculation of plants [**(1AI)** injection, **(1AII)** injury/injection and **(1AIII)** medium culture] and *M. pudica* transferred to the vessel previously *in vitro* infected **(1AIV)**; **(B)** Inoculation of plants in vessel (injection, injury/injection, and soil); **(C)** tests in *Tenebrio molitor*; **(D)** tests in BALB/c mice. ^∗^Inoculum of the studied strains and ^∗∗^all experiments were monitored by the slides and re-isolation in culture in the plate.

Plants of *M. pudica* were inoculated with 10 μL of the strains culture solutions at concentrations of 10^2^, 10^3^, 10^4^, 10^5^, and 10^6^ cells/mL, whereas the plants of *B. gasipaes* were inoculated at concentrations of 10^2^ and 10^5^ cells/mL. Positive and negative controls had equal volumes. The experiments were performed in duplicate with groups of four plants per evaluated strain The inocula were applied at five different points on the plant stem using 10 μL containing 10^2^ cells/mL of each tested strain according to three protocols: (I) direct injection into the stem, (II) stem injury followed by micropipette inoculation, and (III) suspension of culture medium around the root (**Figure 1A**). The plants were maintained *in vitro* under controlled temperature at 28°C. Plant detected with fungal inside the tissues was transferred to the vessel (IV).

### Vessel Plant Inoculation

The *M. pudica* plants previously inoculated *in vitro* were transplanted to vessels with soil substrate (**Figure 1A**) and maintained at a temperature of 30°C with 85% humidity in a Versatile Environmental Test Chamber (VETC; Panasonic MLR-352). Additionally, 61 plants of *M. pudica* obtained by a micropropagation protocol and maintained under aseptic conditions during 60 days were transplanted to the substrate soil containing loam/vermiculite (2:1). In addition, 24 plants of *B. gasipaes* were maintained in soil vessels at a temperature of 30°C with 85% humidity in a VETC at the EMBRAPA forest facility. After 15 days of incubation, inoculations were performed following three protocols: (I) direct injection into the stem, (II) injury associated with injection in the stem, and (III) suspension of 250 mL of liquid culture of each strain tested containing 1 × 10^6^ cells/mL (**Figure 1B**). The experiments were performed in duplicate with groups of four plants per evaluated strain.

### Plant Sample Microscopy

Histological sections were obtained at days 15, 30, 45, and 60 after infection. Plant samples were fixed and stained based on Bernal et al. (2015). The slides were observed and photomicrographs were captured using an Olympus microscope equipped with an SC30 camera.

### Identification of Fungal Colony Recovered From Plant Tissue Artificially Inoculated

The re-isolation of fungi from plant tissues was performed by culturing in Sabouraud’s medium for 2 weeks at 28°C and by flotation technique in mineral oil (Vicente et al., 2008). The fungal ID was confirmed by rDNA internal transcribed spacer (ITS) sequencing (Vicente et al., 2013).

### Virulence Testing With *Tenebrio molitor*

The assessment of survival after fungal infection was done with larvae of *Tenebrio molitor* of approximately 100–200 mg using a total of 10 larvae per strains tested: Fp, Fm and Fe (**Figure 1C**). The inocula of 1 × 10^6^ cells/mL in sterile PBS in aliquots of 5 μL were injected using a Hamilton syringe with a 0.75 mm diameter needle into the hemocoel, the second or third sternite visible above the legs, and the ventral portion. Negative controls included sterile PBS and control sham without physical damage (no treatment). The larvae were placed in sterile Petri dishes and kept in darkness at 37°C. Mortality was monitored once a day for 10 days. The pupae were omitted from the calculation. To detect melanization, the hemolymph of each larva was collected at 4, 24 h, 3, 7, and 10 days postinfection (Scorzoni et al., 2013) and the melanization was determined according to Perdoni et al. (2014). Each hemolymph sample was measured thrice independently. The experiments were performed in triplicate with groups of ten animals, with a total of 30 larvae per group. Survival curves were plotted and statistical analyses were performed according to Maekawa et al. (2015) using the Log-rank (Mantel-Cox), with a *p*-value 0.05 and two-way ANOVA, indicating statistical significance, according to test survival GraphPad Prism 5.

### *Tenebrio molitor* Tissue Burden and Histopathology

Three caterpillars per group (Fp, Fm, and Fe) were weighed and homogenized in 1 mL sterile PBS with a TissueLyser (Qiagen), plated on Mycosel agar, and incubated at 30°C for 14 days. Fungal growth from caterpillars was measured by the number of colony forming units (CFUs) per mL of solution and the re-isolated fungi were sequenced. The samples were embedded in adracanth gum (7 g adracanth in 100 mL of distilled water, 1 drop of formaldehyde), immersed in liquid nitrogen, and sectioned (8 μm) using steel blades in a cryostat (Leica CM 1850). The samples were stained with hematoxylin and eosin (HE) and observed using an Axio Imager Z2 (Carl Zeiss; Jena, Germany) equipped with Metafer 4/VSlide automated capture software (MetaSystems; Altlußheim, Germany) and a CoolCube 1 (MetaSystems) camera.

### Immunogenic Testing in Murine Model

Immunocompetent BALB/c male mice (SPF, 6- to 8-week-old) were maintained under standard laboratory conditions (**Figure 1D**). Inocula of Fp, Fm, and Fe were adjusted to 5 × 10^7^ cells/mL using sterile PBS as the negative control. A volume of 100 μL was injected subcutaneously into the abdomen of the mice. The mice were monitored weekly for up to 30 days postinoculation, on which day histopathological examination of spleen tissue was performed. Western blot analysis was performed using crude protein extracts of the test species obtained by maceration of mycelia using liquid nitrogen based on Almeida et al. (2015). The experiments were performed in triplicate. The animal experiments were performed in accordance with the recommendations on animal welfare by the Institutional Ethics Committee of the Federal University of Paraná (approval certificate no. 1002).

### Murine Tissue Histopathology

Histopathological samples obtained from mice were fixed in ALFAC solution containing 80% ethanol, 40% formalin, and glacial acetic acid for 16 h. Subsequently, the samples were subjected to serial dehydration, embedded in paraffin, sectioned into 5 μm slices, and stained with hematoxylin and eosin (H&E) stain (Cardona-Castro et al., 1996) followed by mounting of the samples with Entellan^TM^. The samples were observed and photomicrographs were captured using an Olympus microscope BX51 equipped with capture software cellSens (2008, Olympus Soft Imaging Solutions, Münster, Germany) and coupled with a camera model DP72.

## Results

### Virulence in the Plant Model

**FIGURE 2:**
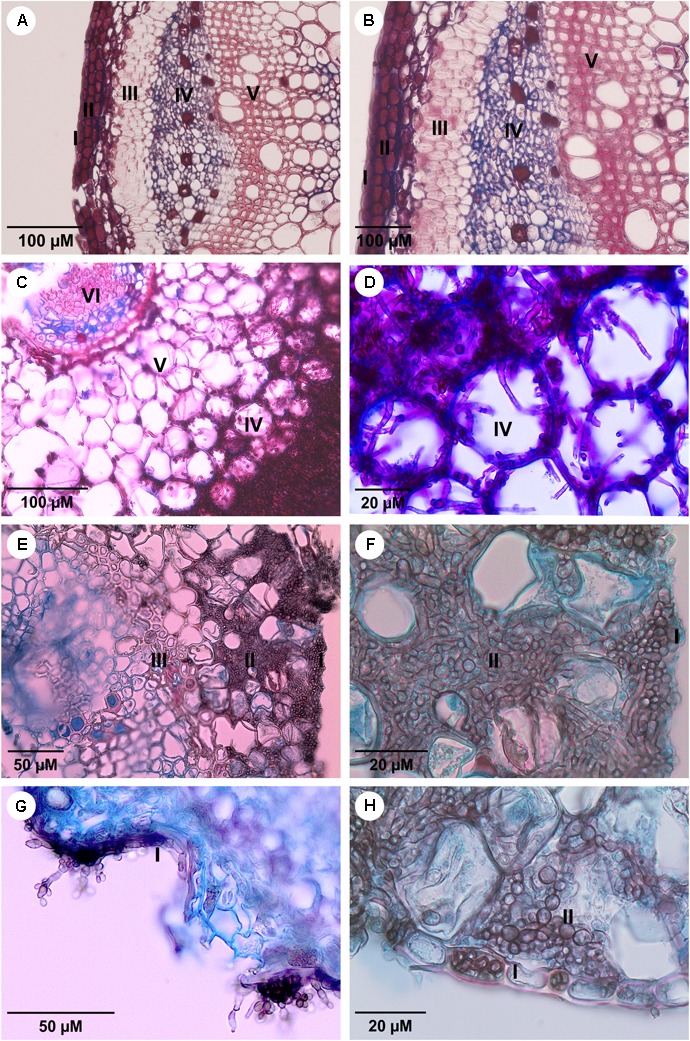
The sections of *Mimosa pudica* micropropagate under histological prudish with Astra Blue and 1% safranin. **(A,B)** Stem histological sections of the control plant without presence of fungus; **(C,D)** root inoculated with *F. erecta* upright; **(E,F)** root inoculated with *F. monophora*; **(G,H)** root inoculated with *F. pedrosoi*. I-Epidermis; II-cortex; III-fiber sheath; IV-phloem; V-xylem; and VI-medulla. The inoculum was applied at five different points on the plant stem using 10 μL with 10^2^ cells/mL of each tested strain.

The environmental species, *F. erecta* and the clinical strains of *F. pedrosoi* and *F. monophora* were inoculated into plant hosts using *M. pudica* and *B. gasipaes*. A total of 152 plants were used, of which 112 were inoculated *in vitro* and 40 were transplanted into the vessels containing autoclaved soil as the substrate. The average height of *in vitro* plants varied from 5 to 12 cm and that of vessel plants varied from 30 to 45 cm. The plants of the *M. pudica* *in vitro* model were small in height, with structures too delicate to injury or injection demonstrating that the infection was evident when conducted by the culture medium inoculation (**Figure 1AIII**). In both plant models, the inocula with densities above 10^2^ cells/mL proved to be quite invasive, leading to weakened and deteriorated plants which did not survive. In other words, at this concentration of the inoculum, all studied fungal strains could grow inside *in vitro* plant tissues (**Figure 2** and **Supplementary Figure S1**), while the host plants did not exhibit any visible external lesions during the assay period.

The *in vitro* plants of *M. pudica* and *B. gasipaes* infected with the clinical species, *F. monophora* and *F. pedrosoi*, presented pseudomycelial cells within the epidermal and cortical tissues, mainly in the intercellular spaces. Hyphae and pseudomycelial cells were concentrated in the parenchyma close to the sclerenchymatic sheath; however, they were absent in the vascular tissue (**Figures 2F,H** and **Supplementary Figures S1H,J**). The invasive property of the fungi was demonstrated by their ability to penetrate the epidermis, reaching the cortical region and consequently leading to the separation of parenchymal cells, with the formation of intercellular spaces that were subsequently colonized by the fungus (**Figures 2E,F,H**). No colonization of the vascular tissue was observed in the plants infected by the strains of the clinical species; reproductive structures of *F. pedrosoi* were observed to grow on the surface of the plant (**Figure 2G**).

In the *in vitro* plants infected with *F. erecta*, smooth, pale brown hyphae with some septa and pseudomycelial cells forming a dense mass in the stalk primary cortex were observed at the location of application of the inoculum (**Figure 2C** and **Supplementary Figure S1F**). The fungus was present inside the vascular tissue, while the endoderm served as an internal barrier (**Figure 2C** and **Supplementary Figure S1F**). The developments were similar in both *M. pudica* and *B. gasipaes* plant models. The endophytic fungi, *Colletogloeopsis dimorpha* and *Cladosporium tenuissimum* utilized as the positive controls in *M. pudica*, and *B. gasipaes*, respectively (**Supplementary Figures S1C,D**), demonstrated similar growth patterns in plant tissues as *F. erecta* (**Supplementary Figures S1E,F**).

Plants of the *in vitro* infected with *M. pudica* demonstrated root invasion by all fungi analyzed after 30 days of culture followed by their transfer to vessels (**Figure 1AIV**). After 60 days, both clinical and environmental strains were observed in the root and stalk tissues; epidermal regions and cortical cells contained large fungal concentrations (**Supplementary Figures S2C,E–G,I**) with the cells of *F. erecta* in the vascular tissue (**Supplementary Figure S2E**).

The plants of *M. pudica* previously produced *in vitro* and transferred to vessels for later inoculation by the injection and/or injury, respectively, (**Figures 1BI,II**) also did not display fungal presence in the histological sections of stalk tissue. In contrast, fungi were observed in the stalk tissues of *B. gasipaes* in the vessels infected by both methods (**Supplementary Figures S3C,E,G,I**).

In the plants of *M. pudica* and *B. gasipaes* produced *in vitro* and transferred to the vessel and infected via soil (**Figure 1BIII**), pseudomycelial cells and hyphae were observed only in the roots of the plants infected by *F. erecta*, which grew as an endophyte similar to the controls (**Supplementary Figures S2D,F**, **S3D,F**). The clinical species were unable to invade the plants by this infection route during the test period of 60 days (**Supplementary Figures S2H,J**, **S3H,J**), implying their ability to invade the plants by this route only during early growth phases via small roots. In contrast, the environmental species could invade the plants through the soil route during all growth phases, indicating a higher ability of invasion and adaptation of these species to living plant tissue (**Supplementary Table S1**)

### Virulence in the *Tenebrio molitor* Larvae Model

**FIGURE 3:**
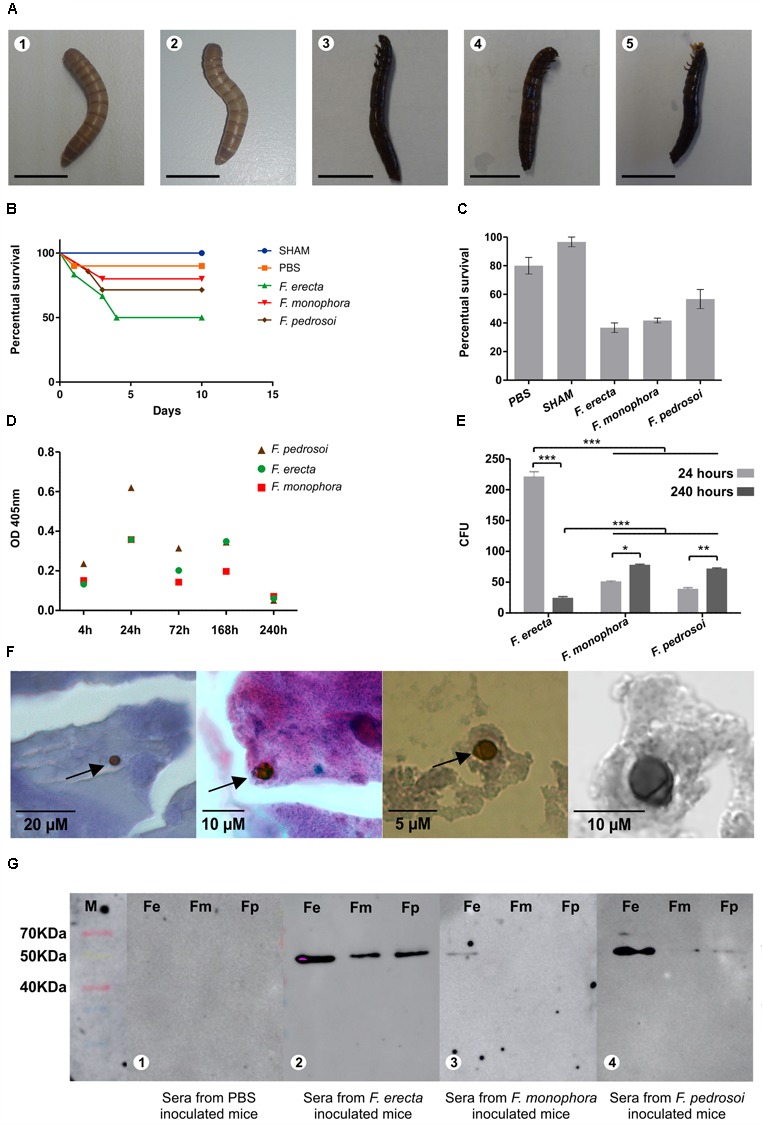
The virulence test using *Tenebrio molitor* and detection of antigenic components using BALB/c mice infected with *Fonsecaea pedrosoi, Fonsecaea erecta*, and *Fonsecaea monophora*; **(A)** inoculated larvae with PBS, SHAM **(1,2)** and infected larvae with *F. erecta* **(3)**; *F. monophora* **(4)**; and *F. pedrosoi* **(5)**. It was used 10 larvae per group; **(B)** the survival curves of the larvae are shown. For each experiment, two different controls were used: untouched larvae (SHAM) and larvae injected with PBS; **(C)** percentage survival of larvae infected with *F. pedrosoi, F. erecta*, and *F. monophora* (Fm) is shown; **(D)** the melanization of the hemolymph is demonstrated by measuring the OD_405_ nm of the hemolymph at 4, 24, 72, 168 and 240 h after infection; **(E)** larvae were homogenized in PBS at 4, 24, 72, 168, and 240 h after infection with *Fonsecaea* for CFU determination; **(F)** occasional muriform cells observed in tissues larvae infected with *F. pedrosoi*; **(G)** Western blot analysis of total proteins from *F. erecta, F. monophora*, and *F. pedrosoi* against serum from mice infected with the same species. Panel **(G)** depicts that a band of approximately 50 kDa was recognized by the sera. Serum was collected from BALB/c mice 30 days postinfection.

This study is the first of *Fonsecaea* virulence testing using *T. molitor* as an infection model. In all species analyzed, the larvae were successfully infected with inoculum concentrations of 1 × 10^6^ cells/mL. During the 10 days of monitoring, the infected larvae displayed lower survival rates than compared to those in the control groups (**Figure 3B**). The larvae infected with *F. erecta* exhibited the lowest survival rates, followed by *F. monophora* and *F. pedrosoi*, demonstrating that all species evaluated were able to survive inside the animal host. The infection rates were significant (*F*: 33.50; d_f_: 4; *p* < 0.01), with larval survival rates ranging from 35 to 69% (control: 5%; **Figure 3C**). According to the histological sections of larvae all species produced yeast cells and hyphae inside the tissues. However, in larvae infected with *F. pedrosoi*, at 120 h postinoculation, was observed spherical or ovoidal cells, 5–20 μm in diameter, with a dark, and thick, multilayered cell wall resembling muriform cells (**Figure 3F**).

Larvae melanization is an intracellular and key defense response to microbial infection. The larvae inoculated with all *Fonsecaea* species presented a gradual increase in the concentration of melanin (pigmentation) within 24 h after inoculation, as assessed both by visual observation and detection of melanization in the hemolymph using spectrophotometry (**Figure 3A**). The pigmentation could be observed during the entire 10-day period of analysis (**Figure 3D**).

The fungal burden inside the larvae was assessed 24 h postinoculation. Significantly higher CFUs of *F. erecta* in the culture medium than that of *F. monophora* and *F. pedrosoi* were observed (**Figure 3E**). The environmental species of *F. erecta* yielded 220 CFUs/mL, while the clinical species, *F. monophora* and *F. pedrosoi* yielded 50 and 38 CFUs/mL, respectively. These results were confirmed by an increase in the number of cells after 72 h, again with *F. erecta* presenting the highest number of 495 CFUs/mL, followed by *F. monophora* with 97 CFUs/mL and *F. pedrosoi* with 101 CFUs/mL. However, after 240 h, the infected larvae that had survived exhibited a significant reduction in the growth of *F. erecta* with 23 CFUs/mL, while those infected with *F. monophora* and *F. pedrosoi* yielded 77 CFUs/mL and 71 CFUs/mL, respectively, suggesting that the environmental species could survive inside the animal (**Supplementary Table S1**) which were demonstrated by the statistical analysis.

### Virulence in Murine Model

The plant-associated species, *F. erecta*, appeared to be more immunogenic in mice than the clinical species, as evident by the recognition of the fungal antigens in the serum of infected mice. The sera obtained from mice 30 days postinfection with *F. pedrosoi, F. monophora*, and *F. erecta* were incubated with the total proteins obtained from the same fungi to detect the antigenic components. In **Figure 3G** depicts that a band of approximately 50 kDa was recognized by the sera. This reaction was more evident when mice were infected with *F. erecta* against all protein extracts. More intense reaction was obtained with *F. erecta* protein extract (**Figures 3G, 4**). At 30 days after inoculation of *F. pedrosoi, F. monophora*, and *F. erecta*, were spleen tissue analysis was performed to check for systemic infection (**Supplementary Table S1**). However, no change in the sectioned spleen tissues was observed (**Supplementary Figure S4**).

## Discussion

The rationale of the present study was to establish the degree to which the etiologic agents of chromoblastomycosis could reside in plant material. *F. pedrosoi* and *F. monophora* are endemic in humid climates (Badali et al., 2008; Najafzadeh et al., 2011; Gomes et al., 2016), particularly tropical areas, such as the Amazon rainforest (Vicente et al., 2013). Two plant hosts with similar distribution were selected. *M. pudica* is a creeping annual or perennial herb belonging to the pea family, *Fabaceae*; it is native to South and Central America and currently considered a pantropical weed that is also prevalent in the Asian countries (Souza Filho et al., 2001). It has been associated with thorn trauma, leading to chromoblastomycosis (Salgado et al., 2004; Salgado, 2010). *B. gasipaes* is a palm native to the tropical forests of South and Central America. It is a long-living perennial plant, which on average is productive for 50–75 years. Species of *Palmaceae* have been reported as a habitat of the melanized fungi (Gezuele et al., 1972; Caligiorne et al., 2005). Silva et al. (1995) reported patients presenting with chromoblastomycosis lesions on the buttocks due to an exposure to palm debris while processing babassu coconuts, a common activity in the Maranhão state of Brazil considered the main endemic area in this country (Gomes et al., 2016).

Our data demonstrated that both clinical and plant-associated *Fonsecaea* species were able to growth inside the two tissues plant models, *M. pudica* and *B. gasipaes* (**Figure 2** and **Supplementary Figure S1**, **S2**, **S3**) using both *in vitro* and vessel plants as hosts. *M. pudica* represents an attractive model for *in vitro* analysis because of its rapid growth. It was invaded when the fungus was inoculated via culture medium *in vitro*; however, the vessel invasion only occurred when it was previously infected *in vitro* (**Supplementary Figures S2C,E,G,I**), although, there are a previously reports of this plant colonized by the fungus related to the disease (Salgado et al., 2004; Salgado, 2010). The fine stems of the plant are difficult to manipulate, which interferes with the *in vitro* inoculation. The palm tree, *B. gasipaes*, our second model, with a long life cycle serves as an excellent model once the plant has a more robust stalk, which makes handling easier; the plant could be infected by different methods of inoculation both in vessels and *in vitro*. Moreover, its fruit pulp was already used as substrate for culture media in order to produce sclerotic cells *in vitro* (Silva et al., 2008). We believe that the method of inoculation may have influenced our results. The observation that the inoculum in the *in vitro* culture medium applied around the root allowed invasion of several tissues in both plants used (**Figure 2** and **Supplementary Figure S1**) could be the way in order to investigate the route of this implantation disease.

The current hypothesis is that patients suffering from chromoblastomycosis acquire the infection via injury from plant material (Queiroz-Telles and Santos, 2013; Queiroz-Telles et al., 2017). This may be considered a possibility; however, it fails to provide the explanation behind the different isolation rates between the species from humans and plant material. The species must differ significantly in predilection for either host (de Hoog et al., 2007; Vicente et al., 2013). According to the comparative genomics of sibling species of *Fonsecaea* associated with human chromoblastomycosis (Vicente et al., 2017) these fungi are able to degrade plant and animal substrates demonstrating a duality in lifestyle that might allow host shifts in Chaetothyriales from environmental niches to animal tissue.

In the present study, we expected low invasion rates of plants by the clinical species and low animal virulence in the plant species. However, we found *F. erecta* to have the largest CFUs in the larvae model suggesting to be the most immunogenic in mice (**Figure 3G**). It was also observed to be the most invasive in the used plant models, where it developed as an endophyte with deep invasion.

The above-mentioned finding led us to formulate an alternative hypothesis to explain the observed etiology of chromoblastomycosis by *Fonsecaea* species (Queiroz-Telles et al., 2017). The clinical species, *F. monophora* and *F. pedrosoi* were seen to remain in the epidermis of the stem in the inoculated plants. The epidermis is responsible for the formation of the thorn, and thus might provide an explanation of the thorn acting as the vehicle of transmission of the “clinical” species, rather than of *F. erecta* that invaded the deeper tissues with a colonizing profile similar to that of the endophyte controls. The larvae initially displayed no defense against this uncommon invader, but later developed an immune response, also observed in murine model (**Figure 3G**), that could control the infection, which was not observed for the clinical species that survived into the host tissues. Besides, we also reported morphologic structures similar to the muriform cells inside the tissues larvae infected by *F. pedrosoi* (**Figure 3F**). Likewise, others studies with animal host, e.g., in mice, already have shown production by muriform cells conidia by *F. pedrosoi* (Sousa Mda et al., 2011; Siqueira et al., 2017; Vicente et al., 2017).

After transcutaneous implantation into a human host, propagules of the agents of chromoblastomycosis become meristematic and form muriform cells (Queiroz-Telles et al., 2017); this is considered to be the pathogenic form of the fungus (Esterre and Queiroz-Telles, 2006). Muriform cells can also be produced *in vitro* by the environmental relatives of the agents of chromoblastomycosis, which has never been reported from this disease (Badali et al., 2008). In the present study, we observed swollen, dark and thick-walled cells that could be considered as a structure similar to the true muriform cells observed in human tissues, where they occasionally have transverse and longitudinal walls.

The mealworm beetle, *T. molitor* is a non-vertebrate animal model that has been used earlier, e.g., in *Candida albicans, Cryptococcus neoformans*, and *Madurella mycetomatis* (Mylonakis et al., 2005; Fuchs et al., 2010; Mowlds et al., 2010; Kloezen et al., 2015; Souza et al., 2015). The larvae infected by *F. erecta* reported the lowest survival rates, but in 240 h, the survived larvae could destroy more *F. erecta* cells than that of *F. pedrosoi*. This suggested that due the fact that *F. erecta* promoted lower survival rates after prolonged periods of infection, higher fungal reduction was achieved, which may be attributed to an increased immune response.

In the virulence test using a murine model was observed that all sera from the infected animals reacted with a 50 kDa molecule (**Figure 3G**). These results indicate that possibly this protein is envolved in the pathogenesis of chromoblastomycosis and it should be better clarified.

In conclusion, the plant infection models employed suggested that all *Fonsecaea* species were saprobic; considering the fact that all agents have to be traumatically inoculated to cause disease, it may be speculated that no primary animal pathogenicity is present in these species. Unexpectedly, *F. erecta* demonstrated to be a virulent species in the animal model, confirming earlier data by Vicente et al. (2017), who concluded the same using *Galleria mellonella* as the animal model. However, *T. molitor* represented a good model to reproduce the disease. The larvae of *T. molitor* have a longer cycle life than those of *G. mellonella*, and we observed for the first time structures similar to muriform cells produced during infection of human tissue in a larvae model. Assuming that all species concerned are opportunists rather than primary pathogens, we might hypothesize that lower virulence is an indication of higher adaptation to the animal host. The results confirm the role of muriform cells as a pathogenic adaptation in animal tissues.

## Author Contributions

GF, VV, SH, and RG conceived and designed the experiments. GF, BS, MV, IB, MN, GS, and BS performed the experiments. GF, VV, RG, JD-G, CB, LM, DK, GS, and BS analyzed the data. JD-G, VV, DS, RS, ET, CA, and MM contributed reagents, materials, and analysis tools. GF, VV, SH, RG, CB, SS, and SA contributed to preparing the manuscript and revising it critically. GF, RG, and VV contributed to annotation and analysis of data; preparation, creation, and/or presentation of the tables, graphics, and figures. VV, CA, ET, DS, AB, SD, CS, and MdS offered strains and/or substantial contributions to the work. VV, RG, SH, SS, and SA conceived and revised the paper. VV, GF, SH, and RG conceived and designed the work and wrote the manuscript.

## Conflict of Interest Statement

The authors declare that the research was conducted in the absence of any commercial or financial relationships that could be construed as a potential conflict of interest. The handling Editor declared a co-authorship with one of the authors SH. The reviewer GB declared a shared affiliation, with no collaboration, with several of the authors, SA and SS, to the handling Editor at time of review.

## Supplementary Material

FIGURE S1 Sections of *Bactris gasipaes* plants under histological prudish with 0.5% toluidine blue and safranin. **(a,b)** Sections of stalk and root in the control plant without the presence of fungus; **(c,d)** the stalk and root inoculated with the positive control (*Cladosporium tenuissimum*) upright; **(e,f)** the stalk and root inoculated with *F. erecta*; **(g,h)** stalk and root inoculated with *F. monophora* (the plant structures and fungus are demonstrated by arrow); and **(i,j)** stalk and root inoculated with *F. pedrosoi*. I-Epidermis; II-cortex; III-fiber sheath; IV-phloem; V-xylem; and VI-medulla.

FIGURE S2 The vessel sections of *Mimosa pudica* under histological prudish with 0.5% toluidine blue and safranin. **(a,b)** Cortes stalk and root of the control plant without the presence of fungus; **(c,d)** stalk and root inoculated with *Colletogloeopsis dimorpha* (positive control); **(e,f)** stalk and root inoculated *F. erecta* (the plant structures and fungus are demonstrated by arrow); **(g,i)** stalk inoculated with *F. monophora* (the plant structures and fungus are demonstrated by arrow) and *F. pedrosoi*; **(h,j)** root of the plant inoculated with *F. monophora* and *F. pedrosoi* without the presence of fungus. I- Epidermis; II-cortex; III-fiber sheath; IV-phloem; V-xylem; VI-medulla.

FIGURE S3 The vessel sections of *Bactris gasipaes* under histological prudish with 0.5% toluidine blue and safranin. **(a,b)** The sections of stem and root in control plant without the presence of fungus; **(c,d)** stalk and root inoculated with *Cladosporium tenuissimum* (positive control); **(e,g,i)** stalk inoculated with *F. erecta, F. monophora*, and *F. pedrosoi*; **(f,h,j)** the plant root inoculated with *F. erecta, F. monophora*, and *F. pedrosoi* without the presence of fungus. I-Epidermis; II-cortex; III-fiber sheath; IV-phloem; V-xylem; and VI-medulla. The plant structures and fungus are demonstrated by arrow.

FIGURE S4 Histologic analysis of the infected tissue of BALB/c with *Fonsecaea* species, the samples were stained with hematoxylin and eosin (HE). **(a,b)** PBS; **(c,d)** *F. erecta*; **(e,f)** *F. monophora*; **(g,h)** *F. pedrosoi*.

TABLE S1 Clinical and environmental strains of *Fonsecaea* sibling species in different infection models.
