# Supplementary figures and images for: A Model for Trans-Kingdom Pathogenicity in Fonsecaea Agents of Human Chromoblastomycosis
Source: Front Microbiol. 2018 Oct 9;9:2211. doi: 10.3389/fmicb.2018.02211 (PMC6189323; doi:10.3389/fmicb.2018.02211)

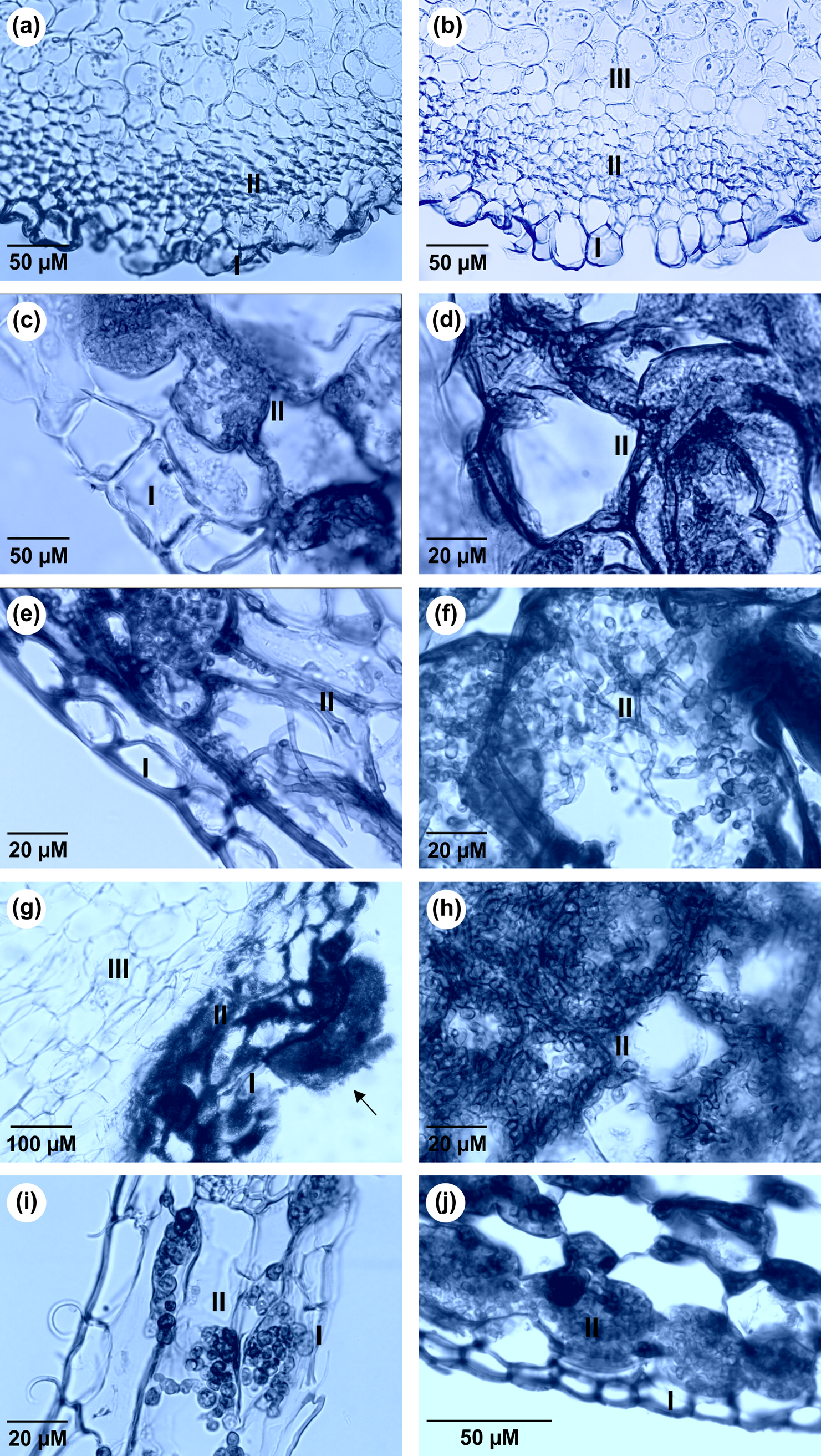

Supplement: FIGURE S1 — Sections of Bactris gasipaes plants under histological prudish with 0.5% toluidine blue and safranin. (a,b) Sections of stalk and root in the control plant without the presence of fungus; (c,d) the stalk and root inoculated with the positive control (Cladosporium tenuissimum) upright; (e,f) the stalk and root inoculated with F. erecta; (g,h) stalk and root inoculated with F. monophora (the plant structures and fungus are demonstrated by arrow); and (i,j) stalk and root inoculated with F. pedrosoi. I-Epidermis; II-cortex; III-fiber sheath; IV-phloem; V-xylem; and VI-medulla. [file Image_1.TIF]

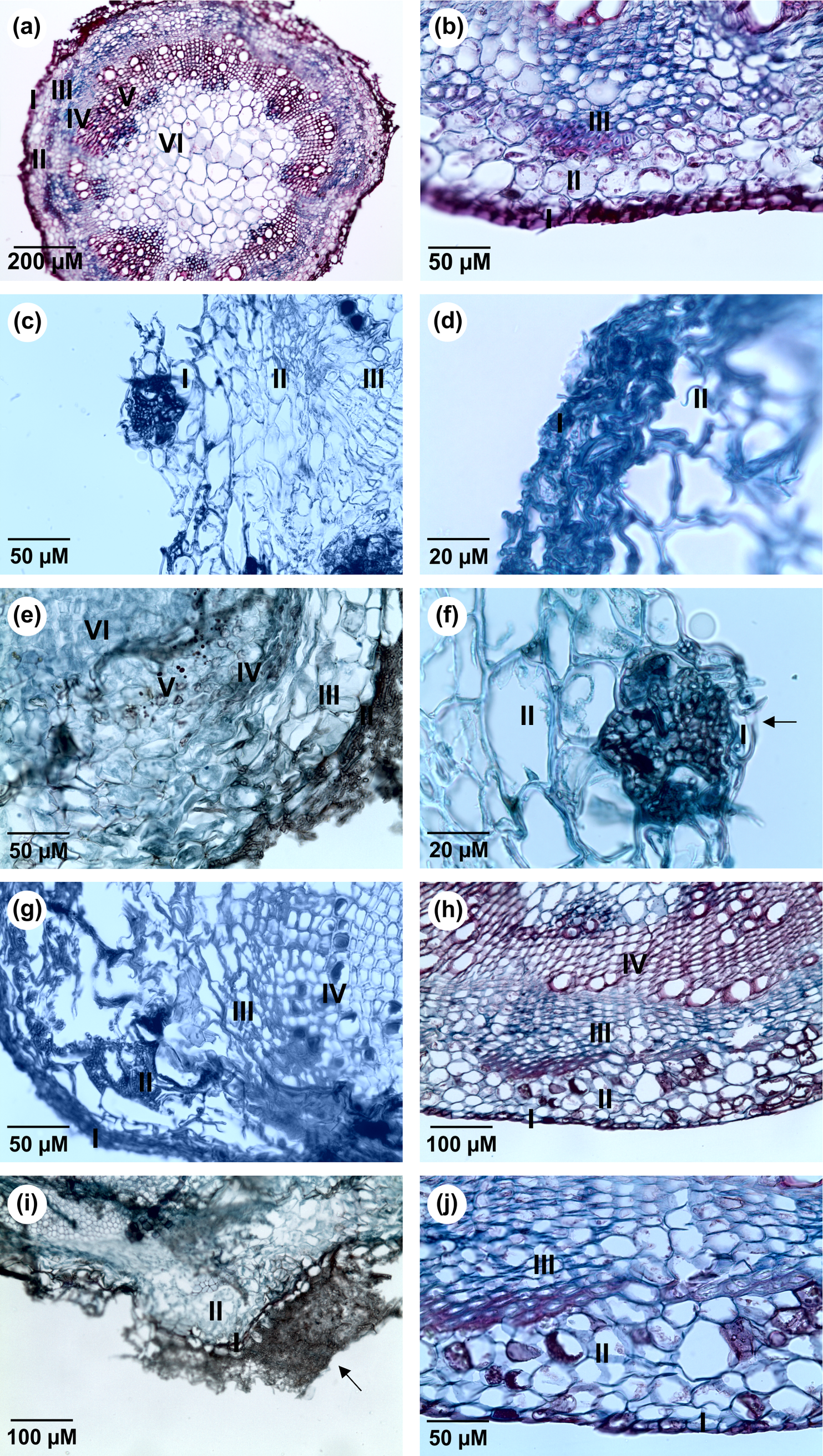

Supplement: FIGURE S2 — The vessel sections of Mimosa pudica under histological prudish with 0.5% toluidine blue and safranin. (a,b) Cortes stalk and root of the control plant without the presence of fungus; (c,d) stalk and root inoculated with Colletogloeopsis dimorpha (positive control); (e,f) stalk and root inoculated F. erecta (the plant structures and fungus are demonstrated by arrow); (g,i) stalk inoculated with F. monophora (the plant structures and fungus are demonstrated by arrow) and F. pedrosoi; (h,j) root of the plant inoculated with F. monophora and F. pedrosoi without the presence of fungus. I- Epidermis; II-cortex; III-fiber sheath; IV-phloem; V-xylem; VI-medulla. [file Image_2.TIF]

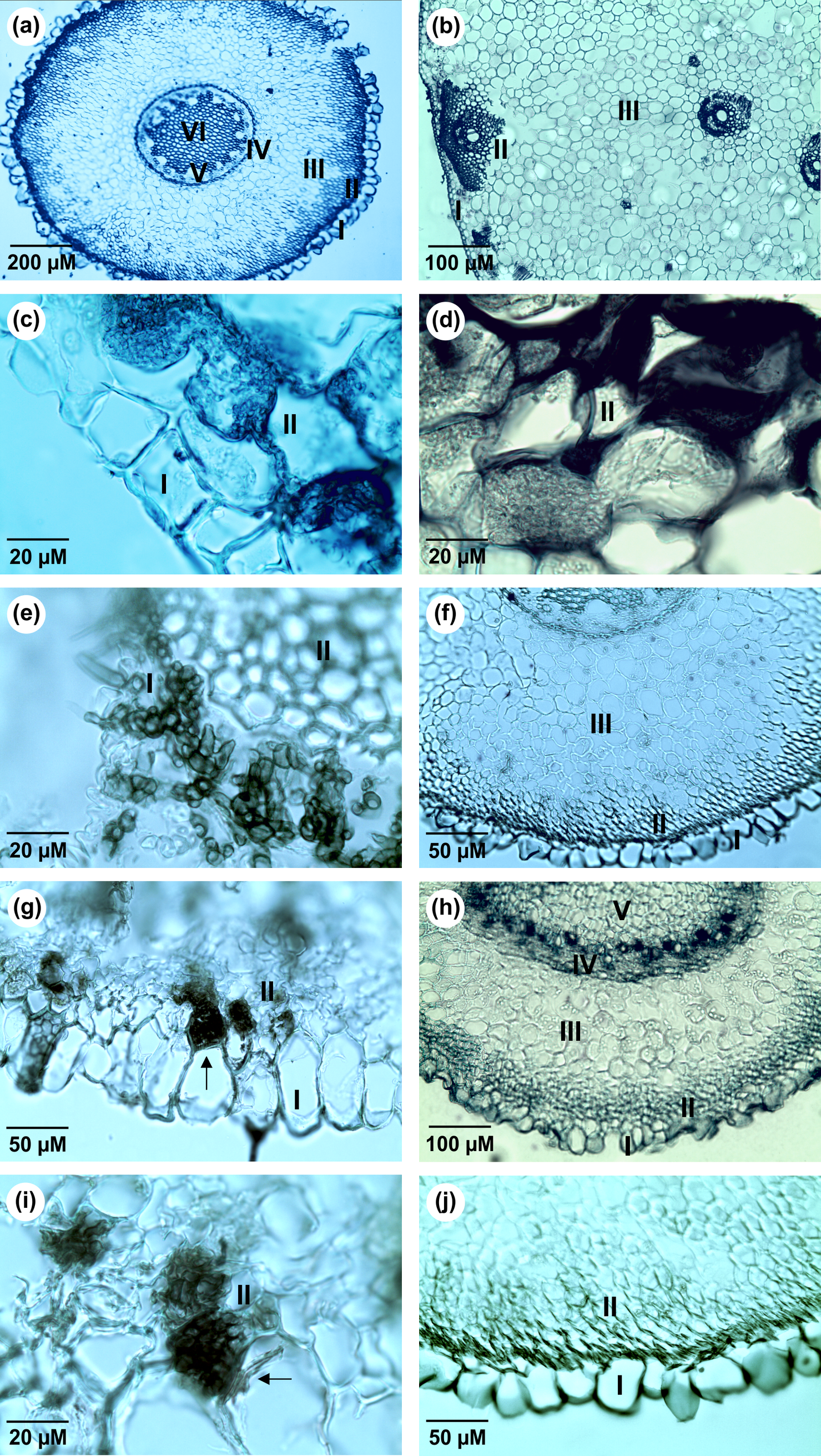

Supplement: FIGURE S3 — The vessel sections of Bactris gasipaes under histological prudish with 0.5% toluidine blue and safranin. (a,b) The sections of stem and root in control plant without the presence of fungus; (c,d) stalk and root inoculated with Cladosporium tenuissimum (positive control); (e,g,i) stalk inoculated with F. erecta, F. monophora, and F. pedrosoi; (f,h,j) the plant root inoculated with F. erecta, F. monophora, and F. pedrosoi without the presence of fungus. I-Epidermis; II-cortex; III-fiber sheath; IV-phloem; V-xylem; and VI-medulla. The plant structures and fungus are demonstrated by arrow. [file Image_3.TIF]

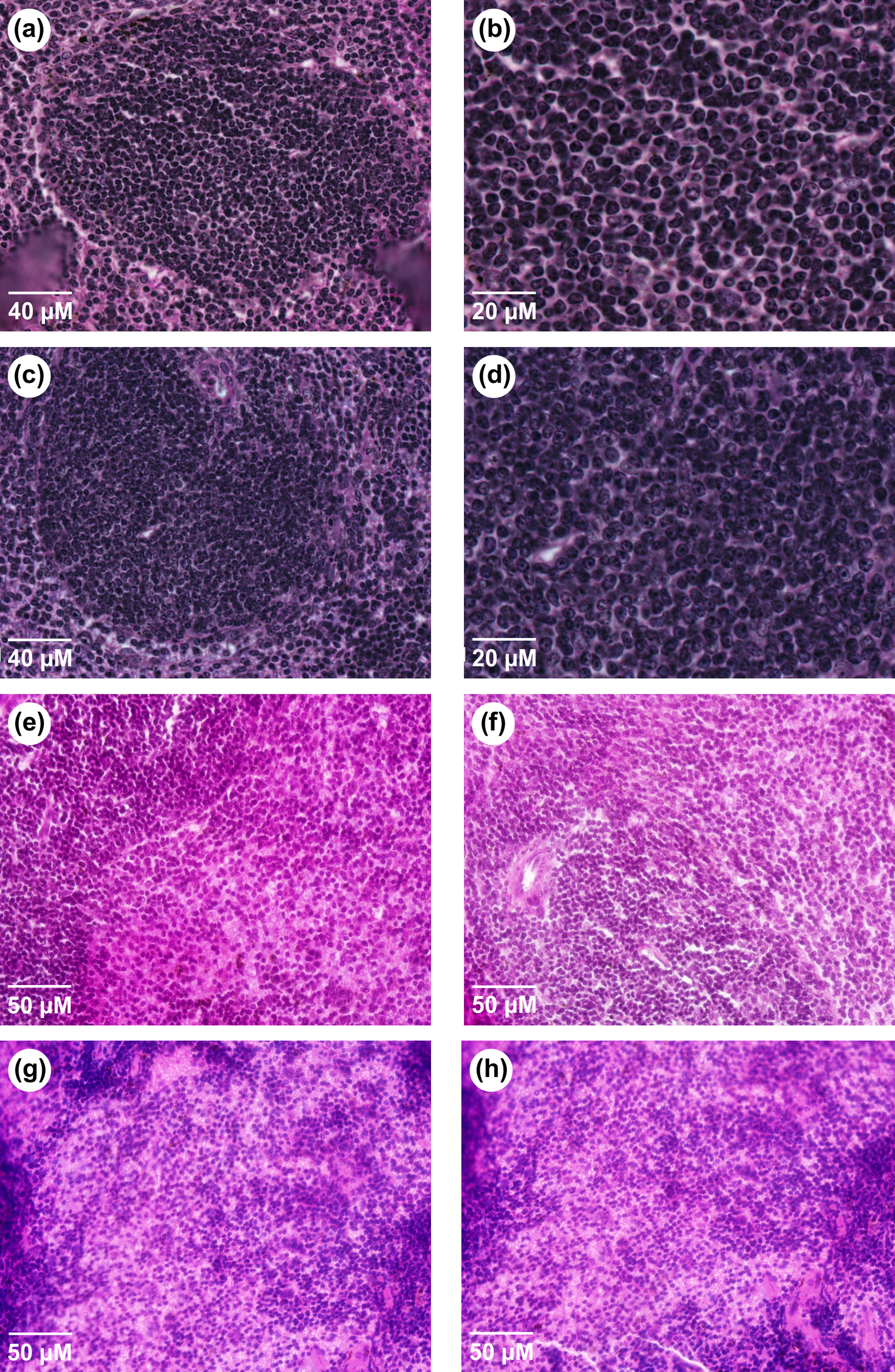

Supplement: FIGURE S4 — Histologic analysis of the infected tissue of BALB/c with Fonsecaea species, the samples were stained with hematoxylin and eosin (HE). (a,b) PBS; (c,d) F. erecta; (e,f) F. monophora; (g,h) F. pedrosoi. [file Image_4.TIF]
