# Supplementary material for: A Model for Trans-Kingdom Pathogenicity in Fonsecaea Agents of Human Chromoblastomycosis
Source: Front Microbiol. 2018 Oct 9;9:2211. doi: 10.3389/fmicb.2018.02211 (PMC6189323; doi:10.3389/fmicb.2018.02211)
Supplement: TABLE S1 — Clinical and environmental strains of Fonsecaea sibling species in different infection models. [file Data_Sheet_1.PDF]

TABLE S1. Clinical and environmental strains of *Fonsecaea* sibling species in different infection models

**Model Assays**	**Clinical Strains**		**Environmental Strains**
**Plants**	***Fonsecaea pedrosoi***	***Fonsecaea monophora***	***Fonsecaea erecta***
In vitro III and IV	Fungal strains were observed in epidermal and cortical tissues in both plants		Fungus was observed in epidermal and cortical to vascular tissues in both plants
In vessel I and II	Fungal strains remained in the epidermal and cortical tissues only in *Bactris gasipaes* palm tree		It was observed from epidermal and cortical to vascular tissues only in *Bactris gasipaes*
In vessel III	By this route plants tissues were invaded only during early growth phases via small roots		Fungal invaded deeper tissues similar to the endophyte species strains used as a control
Tenebrio molitor			
Survival larvae	Higher survival		Lower survival
Fungal burden	Reduced growth at 24 h and increased at 240h		Higher growth in 24h and growth reduction in 240 h
CFU/mL	24h: 38 CFUs/mL<br>240h: 71 CFUs/mL	24h: 50 CFUs/mL<br>240h: 77 CFUs/mL	24h: 220 CFUs/mL<br>240h: 23 CFUs/mL
Histopathology	Presence of hyphae and yeast cells; with swollen dark thick-walled cells resembling muriform cells;	Presence of hyphae and yeast cells	Presence hyphae and yeast cells
**Mice**			
Immunological response	Detected the antigenic protein 50 kDa (+)		Detected the antigenic protein 50 kDa (++)

Legend: Plant inoculation: I-injection; II- injury/injection and III- medium culture inoculation; IV-Plants *in vitro* infected and transferred to the vessel; (+) and (++) represents species immunogenicity.
